# Development of a Cable-Driven Bionic Spherical Joint for a Robot Wrist

**DOI:** 10.3390/biomimetics10010052

**Published:** 2025-01-14

**Authors:** Zixun He, Yutaka Ito, Shotaro Saito, Sakura Narumi, Yousun Kang, Duk Shin

**Affiliations:** 1 Faculty of Engineering, Tokyo Polytechnic University, 1583 Iiyama, Atsugi 243-0297, Kanagawa, Japan; t2806@t-kougei.ac.jp (Z.H.); yskang@t-kougei.ac.jp (Y.K.); 2 Department of Mechanical Engineering, Tokyo Polytechnic University, 1583 Iiyama, Atsugi 243-0297, Kanagawa, Japan; 3 Graduate School of Engineering, Tokyo Polytechnic University, 1583 Iiyama, Atsugi 243-0297, Kanagawa, Japan

**Keywords:** bionic robots, robotic wrists, spherical joints, cable-driven mechanism

## Abstract

Wrist movements play a crucial role in upper-limb motor tasks. As prosthetic and robotic hand technologies have evolved, increasing attention has been focused on replicating the anatomy and functionality of the wrist. Closely imitating the biomechanics and movement mechanisms of human limbs is expected to enhance the overall performance of bionic robotic hands. This study presents the design of a tendon-driven bionic spherical robot wrist, utilizing two pairs of cables that mimic antagonist muscle pairs. The cables are actuated by pulleys driven by servo motors, allowing for two primary wrist motions: flexion–extension and ulnar–radial deviation. The performance Please confirm if the “1583 Iiyama” is necessary. Same as belowof the proposed robot wrist is validated through manipulation experiments using a prototype, demonstrating its capability to achieve a full range of motion for both ulnar and radial deviation. This wrist mechanism is expected to be integrated into robotic systems, enabling greater flexibility and more human-like movement capabilities.

## 1. Introduction

Robots are increasingly integrated into public services and domestic environments, highlighting the importance of bionic design in robotic hand research [1,2]. One of the main challenges for biomimetic robots is accurately replicating the biomechanical properties of human joints [3]. Recent advancements in robotic structures have shown that human-like robot hands exhibit greater dexterity than conventional robots, especially when performing tasks in uncertain environments [4,5,6].

The spatial localization of the hand is crucial for task execution, and almost all robotic hands are equipped with some form of wrist mechanism to support this function [7,8]. However, both academic and industrial research communities have prioritized the development of hand and finger designs, often overlooking wrist systems. It is important to note that the wrist plays a significant role in improving the dexterity of robotic hands by reducing the need for compensatory movements in other joints. This role becomes even more critical when the hands or fingers are simplified or constrained, such as during cylindrical grasping [9].

The human wrist is a highly complex structure comprising bones, ligaments, and redundant tendon systems. These features enable three degrees of freedom (DOFs) and provide exceptional dexterity and functionality. This complexity makes designing a biomimetic wrist particularly challenging. Conventional approaches often simplify the human wrist into mechanical equivalents like hinges, linkages, and gimbals [10,11,12,13,14]. For example, [15] proposed an asymmetrical spherical parallel manipulator for prosthetic wrists, capable of replicating the three primary movements of the human wrist. Experimental results demonstrated that the mechanism could achieve the range of motion (ROM) necessary for daily living activities. However, most of these mechanisms are constructed from metal, making them too heavy for practical use. Shah et al. [16] explored the advantages and disadvantages of a variety of state-of-the-art robotic wrist design solutions, and evaluated their performance in terms of accuracy, workspace, and the complexity of the control method necessary for the system. Investigating methods to optimize the size of a robot while meeting requirements and performance expectations is a critical challenge. Kelaiaia et al. [17] outlined the key challenges in the optimal design of parallel manipulators and proposed a seven-step optimal design method. Robotic joints are often bulkier and less functional than biological joints, making designs that mimic biological structures valuable. A deeper understanding of human motion mechanisms is essential for developing lighter, more compact, and efficient robot wrists [18].

To address the need for greater flexibility, biologically inspired designs have been successfully applied to robot wrists. Scarcia et al. [19] developed a robot wrist capable of mechanically decoupling finger and wrist movements across two DOFs. This design featured two hollow rotational joints with vertical axes, allowing finger-control tendons to pass through without interference. Hyeon et al. [20] replicated the human wrist by simplifying it into two elliptical joints and used shape memory alloys as actuators to achieve motion comparable to that of a human wrist. Kim et al. [21,22] proposed a three-DOF parallel wrist mechanism with a spherical rolling joint supported by three identical links. These links transmitted steering motions to the distal joint, driven by two pairs of cables. Bilancia et al. [23] proposed a robotic compliant wrist incorporating the concept of contact-aided Cross-Axis Flexural Pivots (CAFPs) that successfully mimics human wrist motion and its passive stiffness. While these pioneering efforts have significantly advanced robot wrist research, limitations remain, particularly regarding the size and weight of wrist joints. In addition, most of the current research on robot wrists is not based on human anatomy in their mimicking of human wrists, except for the similarity in tendon actuation mechanisms [10,19,20,21]. These constraints restrict these robots from being applied outside of the laboratory or specialized environments.

While the design of human-like features is very challenging, the benefits of achieving such a design are very significant [4,24,25]. With these human-like features, robots can serve as excellent platforms for testing hypotheses on human movement or replicating forelimb function as a prosthesis. In our previous research, we developed a biomimetic robotic hand based on human anatomy, replicating key physiological features such as bones, ligaments, and tendons [26,27]. By replicating human-like musculoskeletal and tendon drive systems in robots, robots become more powerful and anthropomorphic. Building on this foundation, this study aims to create a robotic wrist system with human-like characteristics. The proposed method allows the robotic wrist system to better mimic the human anatomy in terms of its mechanical structure and actuation method, retaining more biomechanical advantages. This will also facilitate the future integration of this robot wrist into the biomimetic robotic hand to build a human-like forearm system. For this purpose, the wrist mechanism and drive system must also adhere closely to human anatomical principles.

The design requirements for the proposed robot wrist are as follows:

1. Two DOFs to imitate the human wrist: the wrist joint should provide a ROM comparable to that of the human wrist, ensuring sufficient spatial maneuverability.
2. Human-like framework: a human skeletal model should be used as the robot’s framework to achieve a natural appearance and realistic proportions.
3. Cable-driven actuation: a tendon-based cable drive system that replicates the actuation mechanics of a human wrist should be included.

To meet these requirements and address challenges such as mechanical complexity, this study proposes a novel cable-driven robot wrist that combines skeletal and parallel robot design concepts. The robot’s framework is based on a 3D-printed human skeleton model. The wrist bone unit is designed as a socket joint connected to the radius bone via a ball joint for rigid support. The proposed cable-driven spherical parallel joints offer greater flexibility than series-type joint mechanisms. The advantage of employing spherical joints is that the robot’s end-effector can remain coaxial with the wrist and rotate in any direction at a point at a certain distance. This provides the potential to align the robot’s workspace with the actual movement space of the human wrist joint. Unlike conventional parallel robots, which rely on multiple connecting links and universal joints, this design uses cables controlled through pulleys. The proposed robot wrist can mount the actuator away from the joints to reduce the motion inertia of the robot. On the other hand, the cable has excellent flexibility, which can effectively improve the safety of human–robot interaction. Additionally, the tendon displacement mechanism enables high-torque and -speed output while maintaining a compact wrist joint structure.

After the introduction, the rest of the study is organized as follows: Section 2 describes the design of the bionic robot wrist, the inverse kinematics model, and the control system of the robot in detail; Section 3 describes the manipulation experiments we performed on the proposed robot wrist to validate its performance; Section 4 discusses the results of the experiments, and the implications of this study for bionic robotic systems; lastly, Section 5 summarizes the contributions of this study.

## 2. Materials and Methods

### 2.1. Wrist Anatomy

**Figure 1:**
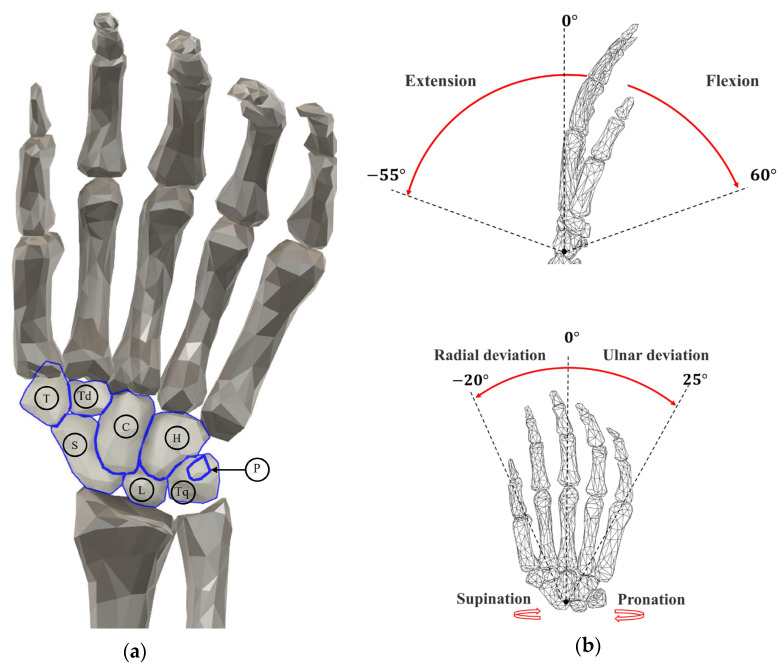
(**a**) The eight carpal bones of the wrist. H: hamate; C: capitate; Td: trapezoid; T: trapezium; P: pisiform; Q: triquetrum; L: lunate; and S: scaphoid. (**b**) The three types of rotational movements of the wrist and the range of movement.

The wrist is one of the most complex joints in the human body, consisting of bones that are tightly bound together by ligaments to provide exceptional flexibility and a wide ROM. As illustrated in Figure 1, the human wrist is composed of eight uniquely shaped carpal bones. The distal ends of the radius and ulna are covered with smooth fibrocartilage, enabling the carpal bones to slide and allowing for flexion–extension (FE) and radial–ulnar deviation (RU) movements [8,28]. Additionally, wrist rotation occurs simultaneously with forearm rotation, as pronation and supination (PS) movements are facilitated by the radius rotating around the ulna.

The maximum ROM for the wrist’s three DOFs is approximately 60°/−55° for FE movements, 25°/−20° for RU movements, and 60°/−40° for PS movements. Most natural wrist movements, however, involve a combination of FE and RU motions. Studies have shown that activities of daily living (ADLs) typically use only 50% to 80% of the wrist’s full ROM [29].

The primary aim of our robotic wrist design is to achieve an anthropomorphic appearance and human-like performance. The proposed robotic wrist incorporates specially designed spherical and socket joints for FE and RU movements. This design avoids the unnatural motion caused by axis misalignment between FE and RU movements while providing a ROM comparable to that of a human wrist joint, as summarized in Table 1.

Considering the weight and size of the actuator, achieving an anthropomorphic design with a human-like appearance would be significantly more challenging if the actuator were placed directly on the wrist. To overcome this limitation, the actuators were relocated on the base platform. This setup simulates tendon actuation by pulling four cables connected to the wrist. The inclusion of redundant actuation in this design allows the stiffness of the joint module to be precisely controlled by adjusting the cable tension, providing greater flexibility in operation. Furthermore, the robot wrist can perform pronation–supination (PS) movements due to a servo motor being connected to the radius bone in series. This feature enhances the potential functionality of the wrist mechanism. However, since the primary goal of this research is to focus on the bionic design of the wrist joint, the PS motion of the robot wrist will not be analyzed or experimentally evaluated in the following sections.

### 2.2. Robot Design

**Figure 2:**
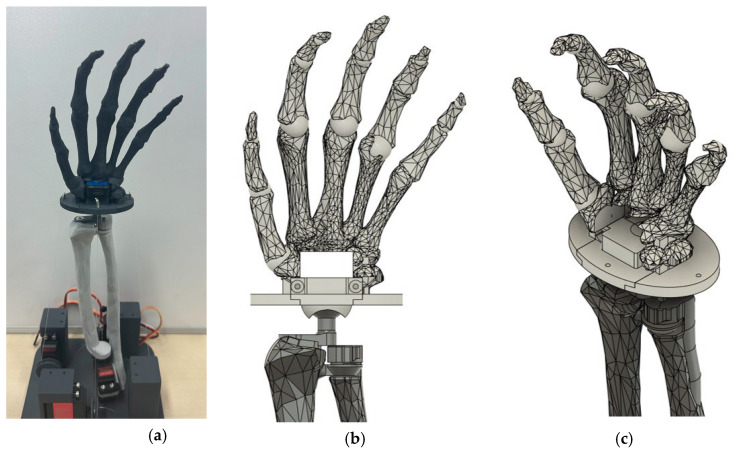
(**a**) Prototype of robot wrist using 3D printing; (**b**,**c**) CAD model of carpal bones and hand.

The prototype of the robot wrist is shown in Figure 2a. To replicate natural human movement, we utilized average bone size data of Japanese individuals, as measured in [30], to create the base model for the robot. After implementing the required design adjustments in CAD software (in Autodesk Fusion 360 v.2.0.20754), the entire forelimb skeleton model, including the phalanges, was fabricated using a 3D printer with PLA material reinforced with carbon fiber.

In the absence of ligamentous tissue to connect the muscles to the bones, we adjusted the positions of the finger bones and securely attached them to their respective metacarpal bones based on the human anatomical structure. A similar approach was applied to the eight carpal bones, completing the hand model shown in Figure 2b,c. A central hole was incorporated into the hand structure to accommodate an IMU sensor for data acquisition.

The spherical joints possess rotational symmetry, allowing rotation about any axis relative to each other [31]. When a gap exists between the socket joint and the ball joint, they can move freely in relation to one another. To simplify the robot’s wrist joint, it was designed as a spherical joint.

In our design, the base of the carpal bone was configured as a socket joint that connects to a spherical component. The gap between the socket and the spherical component was set at 0.5 mm, allowing for smooth movement while maintaining structural integrity. The spherical component was securely screwed to the radius bone to ensure that only rotational movements would occur, providing greater stability.

**Figure 3:**
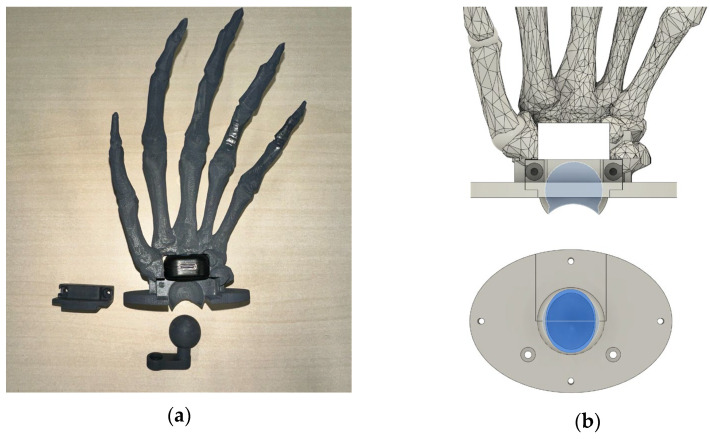
(**a**) The robot wrist was split into two parts and assembled with the spherical joint after the 3D printing of these two parts. (**b**) CAD model of the socket joint.

To limit the ROM of the robot wrist within a predefined range, the socket joint features an oval-shaped cutout, as illustrated in Figure 3b.

The 2-DOF motion of the wrist was achieved by multiple extensor tendon groups being inserted into the carpal bones [32]. However, because of the lack of an ideal method to replicate these connective tissues, a simplified and compromised design was adopted. These tendon groups were reduced to four primary muscles responsible for wrist motion: the flexor carpi ulnaris, extensor carpi ulnaris, flexor carpi radialis, and extensor carpi radialis muscles [32]. A high-tensile-strength fishing line was used to simulate these muscles, maintaining the balance of wrist movements by forming antagonistic relationships.

We designed a small elliptical platform (wrist platform) centered on the spherical joint for transmitting the forces of the cables. Four cables used to simulate tendons were fixed at 90° intervals on the front side of the wrist platform. Cable anchors $A_{1}$ and $A_{3}$ were fixed at $r_{1},r_{3}=$ 20 mm from the ball joint; these drive the wrist platform to perform FE movements. Cables $A_{2}$ and $A_{4}$ were fixed at $r_{2},r_{4}=$ 30 mm from the ball joint; these drive the wrist platform to perform UR movements. These cables pass through the wrist platform and connect to pulleys with a radius of 10 mm. The pulleys, in turn, are controlled by four servo motors mounted on the base platform, ensuring the precise control of wrist movements.

### 2.3. Inverse Kinematics of Robot Wrist

**Figure 4:**
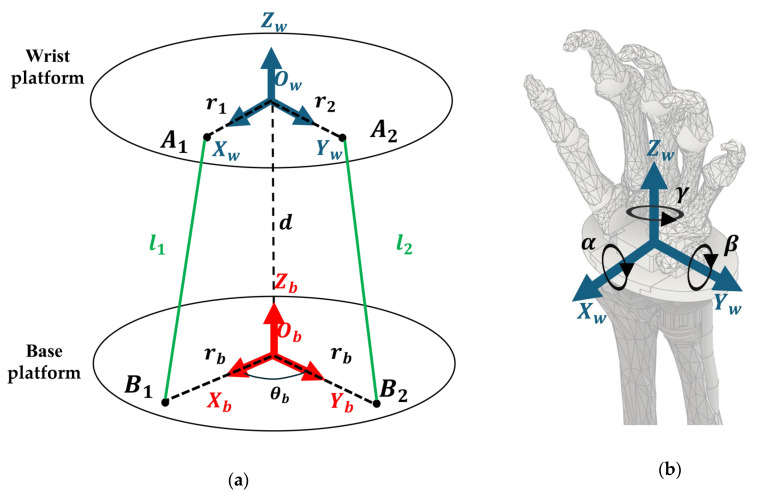
(**a**) Kinematic analysis of the proposed robot wrist mechanism. (**b**) The motion of the wrist joint, represented as rotations of α,β,γ degrees along the $X_{w}$, $Y_{w}$, and $Z_{w}$ axes, respectively.

The proposed robot wrist structure is analogous to that of a spherical–prismatic–spherical parallel manipulator [33]. We can operate the robot wrist by changing the lengths of the cables. To analyze its kinematics, an O–XYZ coordinate system was established for both the wrist platform and the base platform, as illustrated in Figure 4. The center point, $O_{w}$, of the wrist platform is the center of the spherical joint on the wrist. $B_{i}(i=1\;to\;4)$ represents the cable endpoint fixed at the pulley, and crosses the $O_{b}$ point, which is the center of the base platform. The distance between $O_{w}$ and $O_{b}$ is d.

Within this coordinate system, the three primary movements of the wrist joint are as follows: UR movement involves rotation about the $X_{w}$ axis by α degrees; FE movement involves rotation about the $Y_{w}$ axis by β degrees; and PS movement involves rotation about the $Z_{w}$ axis by γ degrees.

We can solve the length of the cable, $l_{i}=\vec{B_{i}A_{i}}$, for a given action by performing an inverse kinematic of the above positional relations. The rotation transformation matrix, $T_{r}$, used to represent the rotational posture of the wrist joint can be expressed as follows:

(1) $$T_{r}=R_{x}\left(\alpha \right)\times R_{y}\left(\beta \right)\times R_{z}\left(\gamma \right)\;R_{x}\left(\alpha \right)=\left[\begin{array}{ccc} 1 & 0 & 0\\ 0 & cos\;\alpha & -sin\;\alpha \;\\ 0 & sin\;\alpha & \cos\;a \end{array}\right]\;R_{y}\left(\beta \right)=\left[\begin{array}{ccc} cos\;\beta & 0 & sin\;\beta \\ 0 & 1 & 0\;\\ -sin\;\beta & 0 & \cos\;\beta \end{array}\right]\;R_{z}\left(\gamma \right)=\left[\begin{array}{ccc} cos\;\gamma & -sin\;\gamma & 0\\ sin\;\gamma & cos\;\gamma & 0\\ 0 & 0 & 1 \end{array}\right]$$

where $R_{x},\;{\;R}_{y},{\;R}_{z}$ in Equation (1) are the rotation matrices around $X_{w}$, $Y_{w}$ and $Z_{w}$ axes of robot, respectively.

The proposed robot only needs to perform FE and UR movements of the wrist, so it is not necessary for it to rotate around the $Z_{w}$ axis; therefore, the vector change of the $Z_{w}$ axis can be ignored. The vector $\vec{O_{b}B_{i}}$ from the center of the base platform, $O_{b}$, to the cable endpoint, $B_{i}$, is as follows:

(2) $$\vec{O_{b}B_{i}}=\left(\begin{array}{c} r_{b}\times \cos\theta_{b}\\ r_{b}\times \sin\theta_{b}\\ 0 \end{array}\right)$$

where $r_{b}$ is the distance from $O_{b}$ to $B_{i}$, and $\theta_{b}$ is the angle between $\vec{O_{b}B_{i}}$ and the $X_{w}$-axis.

Similarly, the vector $\vec{O_{w}A_{i}}$ from the center of the wrist platform, $O_{w}$, to cable anchor $A_{i}$ of the cable in the initial attitude is as follows:

(3) $$\vec{O_{w}A_{i}}=\left(\begin{array}{c} r_{i}\times \cos\theta_{w}\\ r_{i}\times \sin\theta_{w}\\ 0 \end{array}\right)$$

where $r_{i}$ is the distance from $O_{w}$ to $A_{i}$, and $\theta_{w}$ is the angle between $\vec{O_{w}A_{i}}$ and the $X_{w}$ axis.

After the posture of the wrist is changed based on $T_{r}$, the new vector $\vec{O_{w}A_{i}^{l}}$ expressed as follows:

(4) $$\vec{O_{w}A_{i}^{l}}=T_{r}\times \vec{O_{w}A_{i}}$$

Eventually, the length of each cable, $\vec{B_{i}A_{i}}$, after the change in posture is solved by Equation (5):

(5) $$\vec{B_{i}A_{i}}=\left|\vec{O_{w}A_{i}^{l}}-\vec{O_{b}B_{i}}\right|$$

### 2.4. Experimental Setup

**Figure 5:**
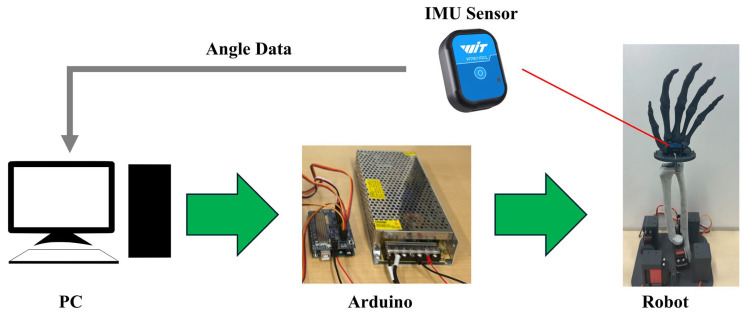
Experimental setup of the robot wrist.

As shown in Figure 5, the experimental setup comprised the prototype of the robot wrist, Arduino, a PC and an IMU sensor. The cable length required to change the posture of the robot was calculated using Equations (1)–(5) in MATLAB. After computation, data were transmitted to Arduino via serial communication to control the servo motor (DS3218MG, Dsservo Technology Dongguan Co., Ltd., Dongguan, China). This motor provides a torque of 20 kg·cm and a 6 V operating voltage is required. To evaluate the robot’s performance, a wireless IMU sensor (WIT9011DCL, WitMotion Shenzhen Co., Ltd., Shenzhen, China) was used to measure the angles of motion, with a sampling rate of 200 Hz. The measured angles were transmitted back to the PC via Bluetooth for further analysis. The robot’s performance was evaluated by root mean square error (*RMSE*), which is defined as follows:

(6) $$RMSE=\sqrt{\frac{1}{N}\sum_{i=1}^{N}{\left(x_{ref,i}-x_{measured,i}\right)}^{2}}$$

where N represents the number of data sets, and $x_{measured,i}$ and $x_{ref,i}$ represent the ith measured trajectory and reference trajectory, respectively.

## 3. Results

Based on the developed robot wrist, two types of trajectory tracking experiments were conducted to evaluate its performance. We wrote a program in MATLAB to execute various wrist movements, including FE, UR, and rotational movements of the wrist. All robot wrist movements were controlled using sinusoidal waveforms with the same phase but differing amplitudes to align with the robot’s ROM, specified in Table 1.

**Figure 6:**
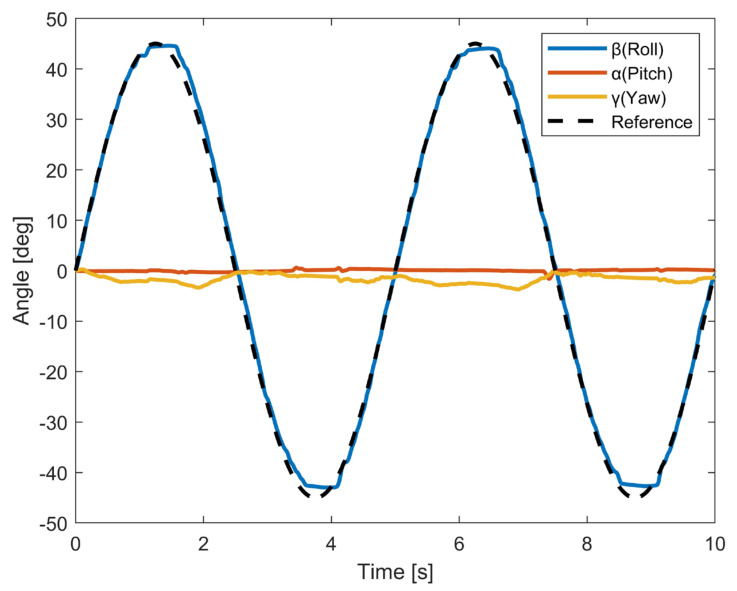
Measured angle data of three dimensions of FE movement. Angle from 0° to 45° correspond to the flexion of the wrist, and those from 0° to −45° correspond to the extension of the wrist.

The results of the FE movements are shown in Figure 6. The proposed robot successfully completed two full cycles of flexion and extension motion. The RMSE between the measured trajectory and the reference trajectory was ${rmse}_{FE}=1.42{}^{\circ}$. During wrist flexion, the wrist angle ranged from 0° to 44.61°, compared with the expected angle of 45°, resulting in an error of 0.39°. During wrist extension, the wrist angle ranged from 0° to −42.98°, while the expected angle was −45°, corresponding to an error of 2.02°. It was observed that there were some displacements in the $Z_{w}$ axis with a maximum angle change of 3.73°.

**Figure 7:**
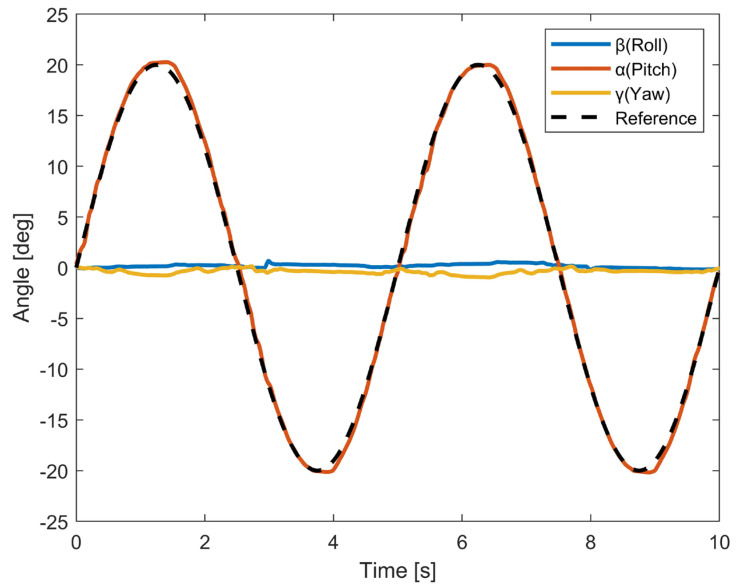
Measured angle data of three dimensions of UR movement. Angles from 0° to 20° correspond to the ulnar deviation of the wrist, and those from 0° to −20° correspond to the radial deviation of the wrist.

The results of the UR movements are shown in Figure 7. The proposed robot was also able complete two cycles of complete ulnar and radial deviation motion, and the RMSE between the measured trajectory and the reference trajectory was ${rmse}_{UR}=0.46{}^{\circ}$. During ulnar deviation, the angle of the wrist ranged from 0° to 20.27°, compared with the expected angle of 20°, resulting in an error of 0.27°. During radial deviation, the angle of the wrist ranged from 0° to −20.18°, compared with the expected angle of −20°, corresponding to an error of 0.18°. It can be observed that there were only small displacements in other axes of less than 1°.

**Figure 8:**
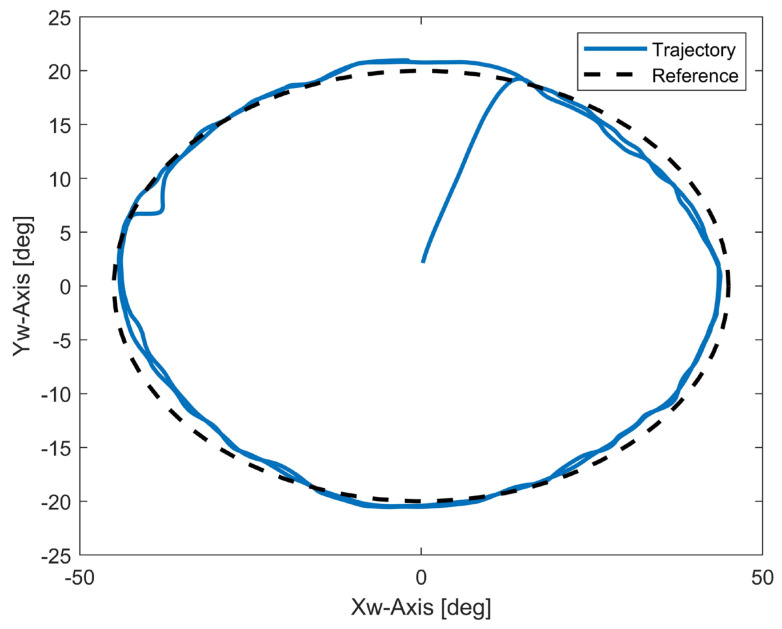
Measured angle data of rotation movements.

Finally, we tested the performance of the proposed robot during rotation movements, and the results are shown in Figure 8. During the rotation movements, the measured trajectory slightly overlapped with the reference trajectory, ${rmse}_{R}=2.67{}^{\circ}$. The maximum error in both the $X_{w}$ axis direction and $Y_{w}$ axis direction during the motion was less than 3°. This demonstrates that the proposed robot can maintain good performance even when executing the two-DOF motions of the human wrist.

## 4. Discussion

In this study, we proposed a novel robot wrist with a spherical joint based on human skeletal anatomy that is capable of smoothly simulating human wrist movements. The robot adopts a modified four-SPS parallel structure, featuring low inertia by positioning all actuators on the base platform. Additionally, we introduced an efficient and robust cable-driven method tailored to the proposed robot’s kinematics. Experimental results confirmed that the proposed robot satisfies our demands. The combination of spherical joints and a cable-driven actuating system enhances the dexterity and versatility of the robot wrist, enabling it to replicate all human wrist movements.

In Figure 6 and Figure 7, we can observe that when the robot was performing single-DOF movement, it was able to efficiently execute the given motion within the designed ROM. Both FE and UR motions have satisfactory trajectory tracking accuracy. And the angle values in other directions are quite stable throughout the motion. We also tested the rotation movements on a robot wrist with a combination of FE and UR motions. Compared with that under single-DOF movement, the trajectory tracking accuracy of the robot was reduced when performing rotation movements. However, it was still able accomplish the task with satisfactory performance. The results demonstrate that the proposed robot wrist can perform all movements of the wrist. One notable advantage of the proposed design is its structural simplicity. The spherical wrist joint consists of a minimal number of parts that can be manufactured using 3D printing. Our proposed bionic spherical joint design significantly reduces the inertia of the robot. Compared to that in previous studies [11,12,13], the proposed design is lighter, is more compact, and can be extended to other joints in bionic robots, such as shoulder and hip joints. Currently, our prototype robot is controlled by two pairs of antagonist muscles. Since the cables are light and can be tightly attached, we can easily increase the number of cables attached to the robot wrist. In future work, we also intend to further extend the achievements of this study to develop an upper-limb robot with a fully human-like redundant system. In order to increase flexibility and ensure performance in human–robot interaction, mapping muscle movements using electromyography (EMG) is an ideal option. For example, muscle activation patterns representing upper-limb motions can be extracted from EMG to estimate the user’s intention and control the robot [34,35].

Despite achieving satisfactory trajectory tracking performance, this research has several limitations. This study was limited to analyzing and testing the robot’s kinematic performance. However, as the proposed robot wrist will be integrated with a bionic hand to create a full upper-limb skeletal robot, it is essential to develop a dynamic model capable of accurately calculating forces and torques at each joint for task execution. Additionally, the coupling issues caused by cable alignment must be addressed to integrate the cable-driven robot wrist into the robot hand [36]. In order to transmit the drive from the base to the rear joint, the cables need to pass through specific paths in the middle joint and be attached to the rear joint. When the middle joint moves, it causes the cable lengths or the tension of the driven cables attached to the rear joint to change, resulting in a relative rotation of the rear joint. In the current design, cables for controlling robot fingers must pass through specific paths on the wrist. When the wrist joint moves, it alters the alignment of these cables, resulting in unintended rotations of the fingers. Future research will focus on methods to solve the motion coupling problem, such as optimizing the structural design and incorporating the closed-loop feedback control of the cable length variation in the control algorithm.

## 5. Conclusions

This study presented a spherical wrist joint designed for bionic robots. The developed robot wrist achieves active two-DOF movements with a wide range of motion, comparable to that of the human wrist. The proposed design addresses the limitations of traditional designs, such as their large size, bulky structures, and restricted mobility. The demonstrated performance of the robot wrist highlights its potential for integration into other robotic systems. Future work will focus on implementing closed-loop control to achieve more precise control and further enhance the performance of the proposed robot wrist.

## Figures and Tables

**Table 1:** Range of motion of human wrist joint and robot wrist.

Human Wrist	Robot Wrist
Flexion: 0 ~ 60°	Flexion: 0 ~ 45°
Extension: 0 ~−55°	Extension: 0 ~−45°
Ulnar deviation: 0 ~25°	Ulnar deviation: 0 ~ 20°
Radial deviation: 0 ~−20°	Radial deviation: 0 ~−20°

## Data Availability

The data and program presented in this study are available on request from the corresponding author.
